# Extracellular macrophage migration inhibitory factor (MIF) downregulates adipose hormone-sensitive lipase (HSL) and contributes to obesity

**DOI:** 10.1016/j.molmet.2023.101834

**Published:** 2023-11-05

**Authors:** Liujun Chen, Lisha Li, Donghong Cui, Yiheng Huang, Haibin Tong, Haleh Zabihi, Shuxia Wang, Yadan Qi, Ted Lakowski, Lin Leng, Suixin Liu, Hong Wu, Lawrence H. Young, Richard Bucala, Dake Qi

**Affiliations:** 1 College of Pharmacy, Rady Faculty of Health Sciences, University of Manitoba, Winnipeg, MB, Canada; 2 Shanghai Key Laboratory of Psychotic Disorders, Shanghai Mental Health Center, Shanghai Jiao Tong University School of Medicine, Shanghai, China; 3 College of Life and Environment Sciences, Wenzhou University, Wenzhou, Zhejiang, China; 4 Department of Cardiology, The General Hospital of Chinese PLA, Beijing, China; 5 Department of Internal Medicine, Yale University School of Medicine, New Haven, CT, USA; 6 Division of Cardiac Rehabilitation, Department of Physical Medicine and Rehabilitation, Xiangya Hospital of Central South University, Changsha, China; 7 Institute of Cardiovascular Disease, Henan University of Chinese Medicine, Zhengzhou, Henan, China; 8 Division of Biomedical Sciences, Faculty of Medicine, Memorial University, St. John's, NL, Canada

**Keywords:** Macrophage migration inhibitory factor (MIF), Hormone-sensitive lipase (HSL), Adipose tissue, Obesity, AMP activated protein kinase (AMPK), c-Jun N-terminal kinase (JNK)

## Abstract

Attenuation of adipose hormone sensitive lipase (HSL) may impair lipolysis and exacerbate obesity. We investigate the role of cytokine, macrophage migration inhibitory factor (MIF) in regulating adipose HSL and adipocyte hypertrophy. Extracellular MIF downregulates HSL in an autocrine fashion, by activating the AMPK/JNK signaling pathway upon binding to its membrane receptor, CD74. WT mice fed high fat diet (HFD), as well as mice overexpressing MIF, both had high circulating MIF levels and showed suppression of HSL during the development of obesity. Blocking the extracellular action of MIF by a neutralizing MIF antibody significantly reduced obesity in HFD mice. Interestingly, intracellular MIF binds with COP9 signalosome subunit 5 (Csn5) and JNK, which leads to an opposing effect to inhibit JNK phosphorylation. With global MIF deletion, adipocyte JNK phosphorylation increased, resulting in decreased HSL expression, suggesting that the loss of MIF's intracellular inhibitory action on JNK was dominant in *Mif*^*−/−*^ mice. Adipose tissue from *Mif*^*−/−*^ mice also exhibited higher Akt and lower PKA phosphorylation following HFD feeding compared with WT, which may contribute to the downregulation of HSL activation during more severe obesity. Both intracellular and extracellular MIF have opposing effects to regulate HSL, but extracellular actions predominate to downregulate HSL and exacerbate the development of obesity during HFD.

## Highlights

• Extracellular MIF binds to CD74 and downregulates HSL through AMPK/JNK signaling.
• Intracellular MIF binds with Csn5 which inhibits JNK phosphorylation.
• With global MIF loss, adipocyte JNK phosphorylation increases, resulting in decreased HSL.
• Both Intracellular and extracellular MIF have opposing effects to regulate HSL.
• Extracellular MIF predominates to downregulate HSL and exacerbate obesity during HFD.

## Graphical abstract

**Figure:**
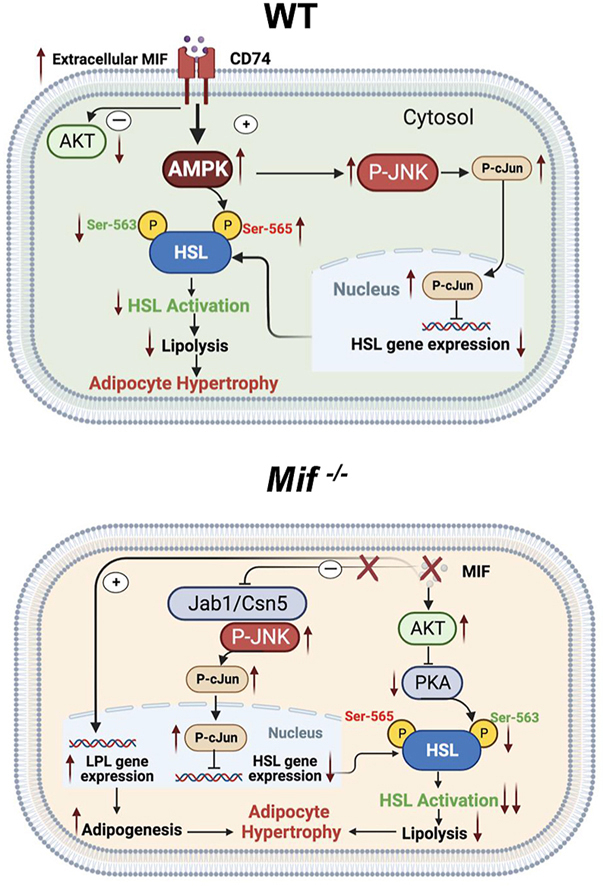

## 1 Introduction

Fatty acid mobilization from adipose tissue is a key mechanism contributing to the development of insulin resistance in the liver and skeletal muscle [1]. Hormone-sensitive lipase (HSL) mediates fatty acid release from adipose tissue by catalyzing hydrolysis of triglycerides and diacylglycerides [2]. Insulin resistance is negatively correlated with HSL gene and protein expression independent of fat mass [3]. Obesity also downregulates HSL activity and norepinephrine-induced lipolysis [4]. The reduction in HSL may contribute to adipocyte hypertrophy and obesity in the setting of HFD and caloric excess. HSL undergoes both transcriptional and non-transcriptional regulation, however, the precise cellular mechanisms underlying the regulation of HSL in obesity are largely unknown.

Metabolic disorders are associated with chronic underlying inflammation in adipose tissue. The cytokine TNF-α has lipolytic actions [5], suggesting that adipose inflammation and inflammatory factors may regulate lipolysis, and endotoxin, which is a potent stimulus of TNF-α release, increased HSL phosphorylation, and stimulated lipolysis in adipose tissue [6]. Thus, classic inflammatory factors appear to regulate lipolysis by activating HSL in adipose tissue.

Macrophage migration inhibitory factor (MIF) is a pro-inflammatory cytokine that upregulates the innate immune response [7]. Circulating MIF levels are also elevated in obese people, while a dietary-focused weight reduction intervention significantly reduces plasma MIF levels [8], suggesting that MIF is positively correlated with adipose tissue mass. Previous studies also have shown that obesity induces adipose MIF expression and cellular release [9]. Our recent work has shown that the antipsychotic olanzapine inhibits HSL and lipolysis in adipose tissue through increasing MIF action and promotes insulin resistance [10]. However, it is currently unknown whether MIF directly downregulates HSL and thus the development of adipocyte hypertrophy during obesity.

MIF has both intracellular and extracellular actions to regulate cell signaling. In immune cells and cardiomyocytes, MIF activates ERK and AMPK signaling pathways through binding with its cell surface receptor, CD74 [[11], [12]]. MIF also exhibits chemokine-like activities through non-cognate interactions with the chemokine receptors CXCR2 and CXCR4 [13]. However, in Hela cells, intracellular MIF directly interacts with Csn5, a coactivator facilitating JNK activation [14]. MIF inhibits Csn5 resulting in the downregulation of JNK signaling pathway [14]. In the present study, we investigated the transcriptional and non-transcriptional effects of extracellular and intracellular MIF in regulating HSL in adipose tissue. We also examined whether MIF inhibition of HSL contributes to lipid accumulation in adipose tissue. Our data suggest that HSL regulation by MIF is an important molecular mechanism that could exacerbate obesity in HFD.

## 2 Materials and methods

### 2.1 Experimental animals

MIF knockout (*Mif*^*−/−*^), CD74 knockout (*Cd74*^*−/−*^), MIF lung transgenic (*Mif* Lung Tg) and wild type littermate (WT) male mice on a pure C57BL/6 background [15,16] were bred at the Health Science Center Animal Facility in Memorial University of Newfoundland or the Animal Care Centre of University of Manitoba, Canada. *Mif*^*−/−*^, *Cd74*^*−/−*^, and their WT littermates at 3 weeks were fed with either normal chow (NC) or high caloric diet (HFD) (#12492, Research Diets, Inc., New Brunswick, NJ, USA) for 12 weeks. *Mif* Lung Tg mice with overexpression of MIF in lung epithelium were developed by Dr. Richard Bucala at Yale University as described previously [16]. These mice have 2-fold increase in *Mif* mRNA expression in their lungs and corresponding increases in production of MIF protein in the bronchoalveolar lavage fluid and alveolar lung epithelium [16]. Currently, *Mif* lung Tg mice are the major MIF overexpression model to study MIF function. There is no evident lung phenotype and the survival rate is normal in *Mif* lung Tg mice [17]. This animal model has normal levels of immune mediators (e.g. TNFα, IL-1beta, IFN-y, leukocytes), wet/dry weight ratios of lungs, arterial oxygen saturation, and serum concentrations of surfactant protein D (SP-D) in the bronchoalveolar lavage compared with WT mice [18]. *Mif* Lung Tg and its littermate were fed with NC for 25 weeks. All experiments were conducted in accordance with the Guide for the Care and Use of Laboratory Animals of the National Institutes of Health and were approved by the Internal Animal Committee Review Board of Memorial University of Newfoundland or University of Manitoba.

### 2.2 MIF neutralization with anti-MIF antibody

As described previously [19], WT Mice (3 weeks) were injected (i.p.) with mouse anti-MIF monoclonal antibody or non-specific IgG at a dose of 20 mg/kg twice a week during high fat diet feeding. Their body weights were monitored. Blood samples and tissues were eventually collected for the further analysis at the end of HFD feeding.

### 2.3 3T3-L1 cell culture

3T3-L1 adipocytes were cultured and differentiated as described previously [20]. Before all experiments, cells were briefly serum-starved in DMEM-0.5 % fetal bovine serum (FBS) for 8 h.

### 2.4 Oil red O staining

Intracellular lipid accumulation was identified by oil red O staining as previously described [21].

### 2.5 The quantification of glycerol and fatty acid release following AICAR or MIF treatment in 3T3-L1 adipocytes

As described previously [22], 3T3-L1 adipocytes were initially washed with PBS to remove the phenol red. Vehicle, AICAR (250 μM), recombinant mouse MIF proteins (400 ng/ml) or isoproterenol (10 μM) was then respectively added to phenol red-free high glucose DMEM supplemented with 2 % BSA for 24 h in the presence or absence of high palmitic acid (100 μM). The culture medium was then collected for the measurements of glycerol and fatty acid by using commercial kits from Sigma (F6428, free glycerol reagent) and Fuji Film (NEFA-HR (2) assay) as per manufacture's protocol.

### 2.6 Co-immunoprecipitation

1 mg/ml of lysed protein was extracted from adipose tissues isolated from WT and *Mif*^*−/−*^ mice. The immunoprecipitation was performed according to the manufacturer's instructions of the Dynabeads™ Protein G Immunoprecipitation Kit (10007D) from Thermo Fisher. Briefly, the Dynabeads-Ab complex was initially prepared by a specific antibody against Jab1/Csn5 (9444, CST) (diluted 1:200) and Dynabeads^TM^ magnetic beads, and then the complex was further mixed with adipose tissue lysates. Following precipitation and elution, the levels of phospho- and total JNK and MIF were analyzed by Western blot [23].

### 2.7 MIF and AMPK knockdown by siRNA

To temporarily silence MIF and AMPK expression in 3T3-L1 adipocytes (cultured in 12-well plates), 1 nM of *Mif* siRNA (*Mif* siRNA, N405895, ThermoFisher) or AMPK Alpha 1/2 siRNA (sc-45313, Santa Cruz) or non-silencing control siRNA (AM4611, ThermoFisher) was transfected into the cells by using INTERFERin® Transfection Reagent (2 μl, Ref#101000016, Polyplus Transfection) in medium without FBS and antibiotics as recommended by the manufacturer for 36 h.

### 2.8 Antibodies and reagents

Antibodies against phospho-AMPK (Thr^172^), phospho-Akt (Ser^473^), phospho-HSL Ser^563^, phospho-HSL Ser^565^, phospho-PKA Thr^197^, phospho-CREB Ser^133^, phospho-JNK, phospho-c-Jun and total AMPK, PKA, Akt, CREB, Csn5, JNK and HSL were purchased from Cell Signaling. Recombinant mouse MIF was purified from a high yield *E. coli* expression system by fast protein liquid chromatography (FPLC) followed by C8 chromatography to remove endotoxin [24]. Mouse MIF concentrations were measured by a one-step sandwich enzyme-linked immunosorbent assay as previously described [25].

### 2.9 Expression analyses

Transcript levels for the mouse genes of *ATGL*, *PPARγ*, *Cd36*, *PPARα*, *CPT-1*, *Tnfa*, *Il1b*, *Il6*, *FASN* and *HSL* (Supplementary Table 1) were measured by qPCR [10]. Phosphorylation and/or total levels of AMPK, Akt, CREB, JNK, c Jun, PKA and Csn5 in adipose tissue or cells were evaluated by Western blot.

### 2.10 Histology

Hematoxylin–eosin (HE) staining was performed to identify adipocyte hypertrophy in adipose tissue as described previously [10].

### 2.11 Statistical analysis

One-way ANOVA with Tukey's post-hoc tests or student t-test was used to determine differences between group mean values. The level of statistical significance was set at P < 0.05.

## 3 Results

### 3.1 The activation of AMPK inhibits HSL and lipolysis through JNK in adipocytes

**Figure 1:**
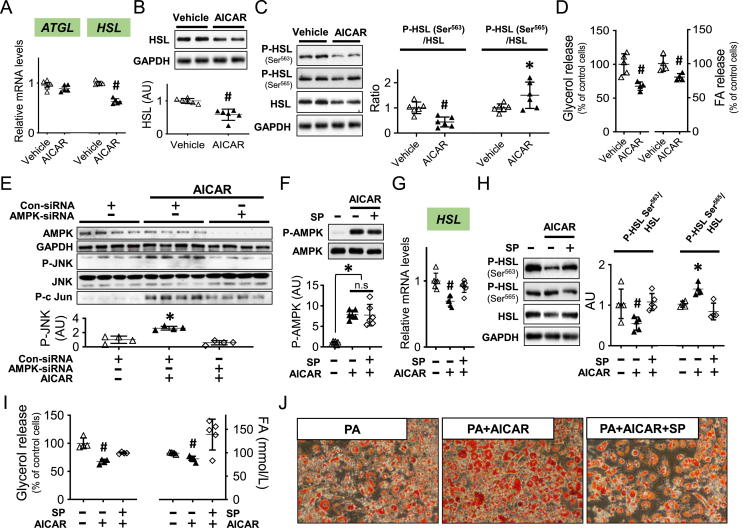
***AMPK activation inhibits HSL and lipolysis through JNK in adipocytes****.* 3T3-L1 cells were differentiated and incubated with an AMPK activator, AICAR (250 μM) for 24 h. *ATGL* and *HSL* gene expression was quantified with qPCR (A). Total and phospho-HSL protein levels were measured with western blot (B and C). Glycerol and fatty acid (FA) release was evaluated in (D). Following knockdown of *AMPKα1* and *α2* isoforms by *siRNA*, AMPK, phospho- and total JNK, and p-c Jun were examined in adipocytes following vehicle and AICAR treatment (E). In a separate experiment, the JNK inhibitor, SP600125 (10 μM) was incubated with AICAR and AMPK phosphorylation, *HSL* gene and protein expression and HSL phosphorylation were subsequently evaluated from (F) to (H). Glycerol and FA were quantified in (I). In the presence of high palmitic acid (PA, 100 μM), Oil red O staining was performed to evaluate lipid accumulation in adipocytes following AICAR with or without SP600125 treatment. A-D were analyzed by 2-tailed Student's *t* test and the rest of the data in addition to J were analyzed by 1-way ANOVA. All data are Mean ± SD. ∗P ≤ 0.05 increase and ^#^P ≤ 0.05 reduction vs. Vehicle in A-D and F; vs. other groups in E, G-I. n.s. represents no significance.

MIF activates AMPK in the heart and liver that promotes fatty acid oxidation [26,27]. However, AMPK also inhibits lipolysis by downregulating HSL activation in adipose tissue [28]. Normally, HSL phosphorylation at Ser^563^ via PKA stimulates HSL activity whereas AMPK phosphorylates HSL at Ser^565^, which leads to reduced phosphorylation of Ser^563^ and lipolysis [28]. In differentiated 3T3-L1 adipocytes, we found that AMPK activation induced by AICAR treatment (250 μM, Fig. S1A) was associated with a reduction in HSL gene and protein expression (Figure 1A–B) but did not change the expression of adipose triglyceride lipase (*ATGL*) (Figure 1A). In parallel, AICAR also upregulated the inhibitory phosphorylation of HSL at Ser^565^ (Figure 1C). These effects of AMPK activation together reduced activating phosphorylation of HSL at Ser^563^ (Figure 1C), were associated with a decrease in glycerol and fatty acid release from adipocytes (Figure 1D). The results suggest that both transcriptional and non-transcriptional effects of AMPK mediate the inhibition of HSL activation which downregulates adipocyte lipolysis.

AICAR also upregulated phosphorylation of JNK and its downstream protein, c-Jun, and these effects were inhibited by AMPK-siRNA, indicating that this action was mediated by AMPK (Figure 1E). However, JNK inhibition by SP600125 did not affect AMPK activation by AICAR (Figure 1F) but prevented the effects of AICAR on HSL expression and phosphorylation (Figure 1G–H) and adipocyte lipolysis (Figure 1I). The AMPK/JNK regulated HSL signaling pathway occurred without changes in PKA signaling pathway (Fig. S1B). In the presence of high fatty acid (100 μM palmitic acid), AICAR augmented lipid storage and this effect was inhibited by SP600125 (Figure 1J). These data together suggest that AMPK activation increases adipocyte hypertrophy through its inhibitory effects on HSL expression and activation.

### 3.2 Extracellular MIF downregulates lipolysis through the AMPK/HSL pathway which facilitates lipid storage in adipocytes following high palmitic acid treatment

**Figure 2:**
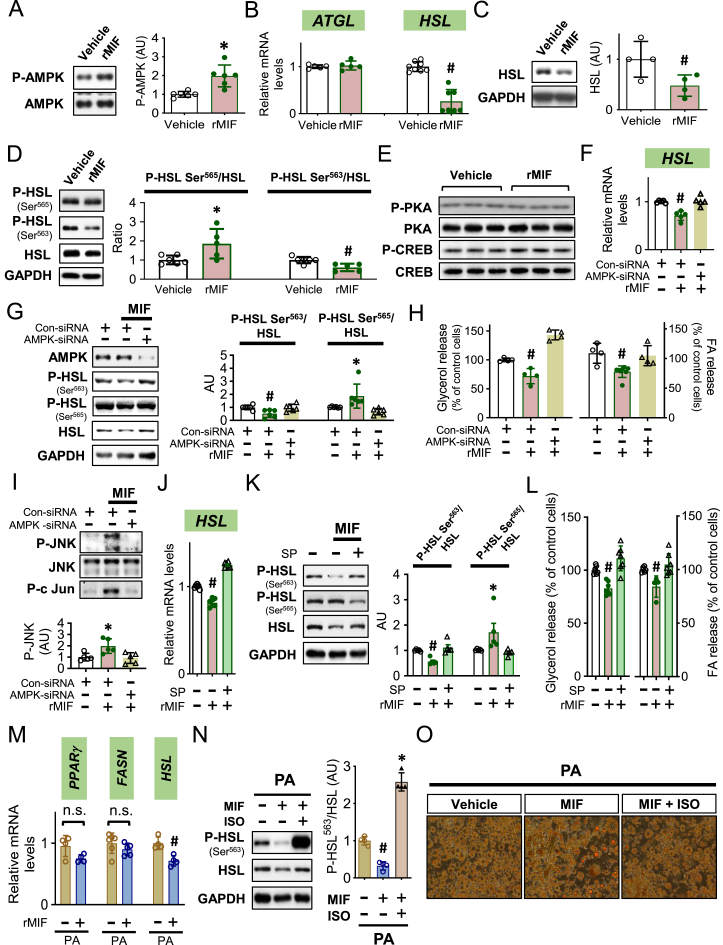
***Extracellular MIF downregulates lipolysis through the AMPK/HSL pathway which facilitates lipid storage in adipocytes following high palmitic acid treatment****.* Differentiated 3T3-L1 adipocytes were incubated with recombinant mouse MIF protein (rMIF, 400 ng/ml) for 24 h. The phosphorylation of AMPK (A), *ATGL* and *HSL* gene (B), and HSL protein (C) expression was evaluated subsequently with qPCR or western blot. Furthermore, the phosphorylation of HSL at Ser^565^ and Ser^563^ sites was assessed by western blot (D). The activation of HSL upstream enzyme, PKA and its downstream, CREB was quantified in (E). Following knockdown of *AMPKα1* and *α2* isoforms by *siRNA*, HSL expression and activation were measured in (F) and (G). The relevant glycerol and fatty acid (FA) release was quantified in (H). In the absence of AMPK, phospho- and total JNK were evaluated by western blot (I). In addition, HSL expression and phosphorylation were quantified by qPCR or western blot following MIF treatment with or without SP600125 (SP) incubation (J and K). Glycerol and FA were also measured in the medium (L). In the presence of high palmitic acid (PA), *PPARγ*, *FASN* and *Hsl* gene was quantified following MIF treatment (M). Isoproterenol (ISO) regulated HSL phosphorylation and lipid accumulation following MIF was examined by western blot and Oil red O staining in (N) and (O), respectively. A-D and M were analyzed by 2-tailed Student's *t* test and the rest of the data in addition to O were analyzed by 1-way ANOVA. All data are mean ± SD. ∗P ≤ 0.05 increase and ^#^P ≤ 0.05 reduction vs. Vehicle in A-D and M; vs. other groups in F-L and N. n.s. represents no significance.

Our previous studies indicated that MIF regulates metabolism through activating AMPK signaling pathway in the heart and hypothalamus [10,11]. In our present experiments, MIF (400 ng/ml) addition to 3T3-L1 adipocytes for 24 h stimulated AMPK phosphorylation (Figure 2A) and decreased HSL gene and protein expression (Figure 2B–C), without changes in the expression of the lipolytic enzyme ATGL (Figure 2B). MIF treatment-activated AMPK also triggered inhibitory phosphorylation of HSL, thus contributing to an attenuation in HSL activation (Figure 2D). MIF also directly inhibited Akt phosphorylation in 3T3-L1 adipocytes (Fig. S2A). However, the downregulation of Akt was not associated with any change in phosphorylation of PKA or its downstream protein CREB (Figure 2E), suggesting that MIF regulation of HSL is independent of the traditional Akt/PKA signaling pathway. MIF treatment also downregulated glycerol and fatty acid release from the cells (Figure 2H), suggesting an inhibitory effect of MIF on lipolysis. After silencing AMPK in adipocytes (Fig. S3), MIF failed to inhibit either HSL expression or activation (Figure 2F–G) and MIF-downregulation of glycerol and fatty acid release was also blocked (Figure 2H). These data confirmed that MIF-mediated HSL and lipolysis are AMPK-dependent.

We found that MIF stimulated phosphorylation of JNK and c-Jun was also inhibited by AMPK siRNA (Figure 2I). JNK mediates the effects of MIF on the expression as well as the activation of HSL and adipocyte lipolysis (Figures 2J-L), independent of the PKA signaling pathway (Fig. S2B). To test whether MIF downregulation of HSL and lipolysis could augment lipid storage in adipocytes in the presence of high fatty acid, we incubated 3T3-L1 adipocytes with MIF in the presence of high palmitic acid (PA, 100 μM) for 24 h. Adipogenesis genes, such as *PPARγ* and fatty acid synthase (*FASN*), were unchanged, but *HSL* gene expression and phosphorylation were lower following treatment with MIF and high PA (Figures 2M−N). In parallel, we also observed enlarged intracellular lipid droplets following MIF treatment (Figure 2O). Isoproterenol increased HSL activation, and successfully reversed the downregulation of HSL by MIF (Figure 2N). As a result, isoproterenol significantly reduced MIF-induced enlargement of lipid droplets (Figure 2O), suggesting a key role of HSL in regulating adipocyte hypertrophy following MIF treatment.

### 3.3 High plasma MIF induces adipocyte hypertrophy and obesity through activating the AMPK/JNK signaling and inhibiting HSL

**Figure 3:**
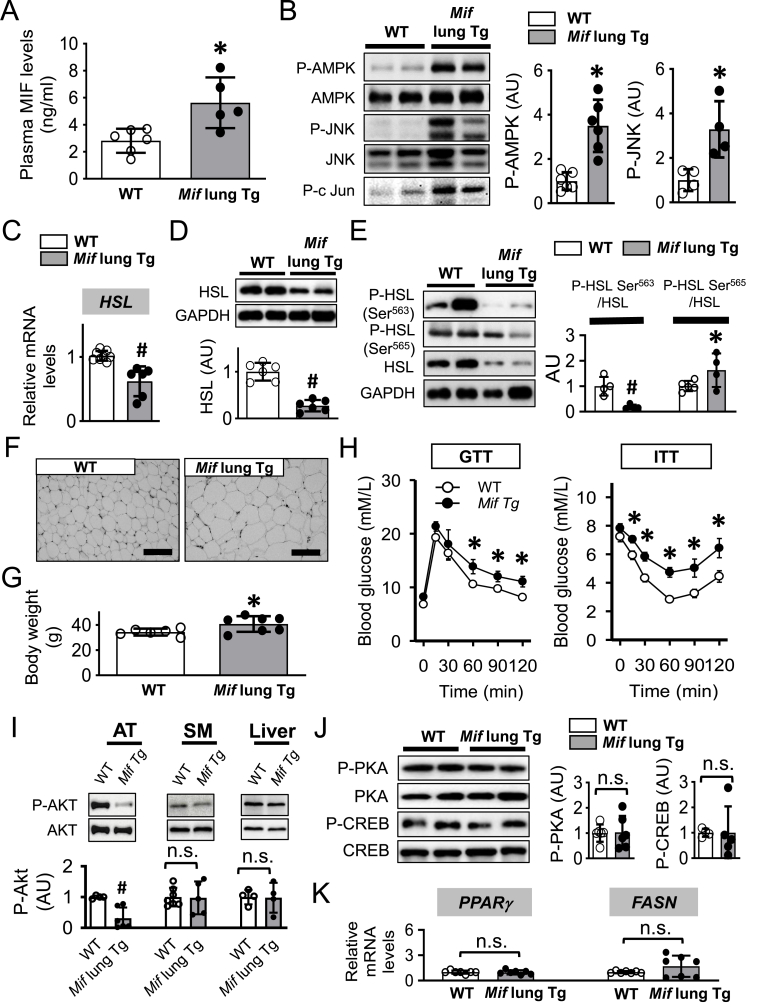
***High plasma MIF induces adipocyte hypertrophy and obesity through activating the AMPK/JNK/HSL signaling pathway****.* Age-matched WT and *Mif* lung Tg mice were euthanized for the quantifications of plasma MIF levels (A), AMPK and JNK activation (B), HSL expression (C and D) and phosphorylation (E), and adipocyte size (F) and body weight (G). Whole-body insulin sensitivity was evaluated by glucose tolerance test (GTT) and insulin tolerance test (ITT) (H). The phosphorylation of Akt in adipose tissue (AT), smooth muscle (SM) and liver was measured in (I). Phosphorylation of PKA and CREB (J), and gene expression of *PPARγ* and *FASN* (K) were evaluated by western blot and qPCR, respectively. H was analyzed by multivariate (2-way) ANOVA and the rest of the data in addition to F were analyzed by 2-tailed Student's t test. All data are presented as mean ± SD. ∗P ≤ 0.05 increase vs. WT in A, B, E, G and H. ^#^P ≤ 0.05 reduction vs. WT in C, D and I. n.s. represents no significance.

To further examine whether the MIF/AMPK/HSL signaling pathway contributes to the development of adipocyte hypertrophy and obesity *in vivo*, we utilized a MIF overexpression model, with transgenic over-expression of MIF (*Mif* lung Tg mice) leading to chronic elevation of circulating MIF. At 25 weeks, these mice had high circulating MIF levels (Figure 3A), which were associated with increased phosphorylation of AMPK, JNK and c-Jun (Figure 3B) and decreased *HSL* expression and activation (Figure 3C–E) in adipose tissue. These mice displayed enlarged adipocyte size (Figure 3F) and increased body weight gain (Figure 3G) compared to age-matched WT mice. We also demonstrated whole-body and adipose-specific insulin resistance in the *Mif* lung Tg mice (Figure 3H–I). Although Akt phosphorylation was attenuated in adipose tissue from these mice, insulin resistance was not associated with any changes in PKA signaling pathway (Figure 3J). In addition, the high plasma MIF levels did not alter adipogenesis gene *PPARγ* and *FASN* expression (Figure 3K), suggesting that MIF-mediated AMPK/JNK activation in adipose tissue selectively impacted HSL and lipolysis during the development of adipocyte hypertrophy and obesity.

### 3.4 High caloric diet upregulates plasma MIF that activates adipose AMPK/HSL signaling and adipocyte hypertrophy

**Figure 4:**
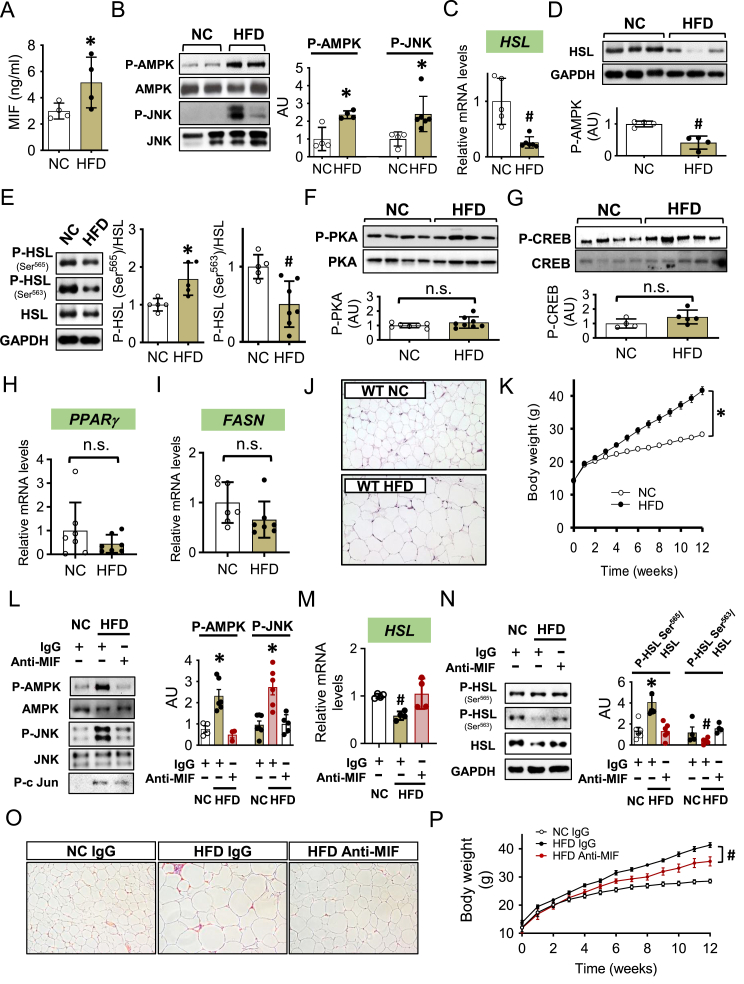
***High caloric diet upregulates plasma MIF that activates adipose AMPK/HSL signaling pathway and augments adipocyte hypertrophy****.* WT mice at 3 weeks were fed with normal chow (NC) or a high caloric diet (HFD) for 12 weeks. Plasma MIF level (A) was measured subsequently. The phosphorylation of AMPK (B), JNK (B), and *HSL* gene (C) and protein (D) expression were quantified in adipose tissue by western blot or qPCR. The phosphorylation of HSL at Ser^565^ and Ser^563^ was evaluated in (E). The phosphorylation of PKA and CREB was also examined by western blot (F and G). The gene expression of *PPARγ* and *FASN* was evaluated by qPCR (H and I). Hematoxylin-eosin (HE) staining was performed to identify adipocyte hypertrophy in (J), and body weight gain was monitored weekly (K). In a separate experiment, non-specific IgG or anti-MIF antibody (20 mg/kg, i.p. twice a week) was injected twice per week accompanied with high fat diet. Phospho- or total AMPK and JNK and HSL expression and phosphorylation were evaluated in (L to N). Adipocyte size was detected by HE staining and body weight gain was monitored and shown in (O and P). A-I were analyzed by 2-tailed Student's *t* test, K and P was analyzed by multivariate (2-way) ANOVA and the rest of the data in addition to J and O were analyzed by 1-way ANOVA. All data are presented as mean ± SD. ∗P ≤ 0.05 increase and ^#^P ≤ 0.05 reduction vs. NC in A-K; vs. other groups in L-N; vs. HFD IgG in P. n.s. represents no significance.

High fat diet (HFD) induces a high level of circulating MIF in animal models [29]. In order to determine whether HFD activates the MIF/AMPK/JNK anti-lipolytic pathway, we fed C57BL/6 mice with HFD (Fig. S4A) for 12 weeks to upregulate plasma MIF levels (Figure 4A). The HFD-induced a two-fold increase in plasma MIF (similar to the *Mif* lung Tg model), which was also associated with higher AMPK and JNK phosphorylation (Figure 4B) and lower *HSL* gene and protein expression (Figure 4C–D) compared to the normal chow (NC) control group. Furthermore, adipose tissue isolated from the HFD group showed increased HSL Ser^565^ and reduced HSL Ser^563^ phosphorylation (Figure 4E). HFD also induced whole-body insulin resistance (Fig. S4B) and attenuated Akt phosphorylation in peripheral tissues, including adipose tissue (Figs. S4C-E). However, similar to our prior observations in the *Mif* lung Tg model, the alterations in adipose HSL expression and phosphorylation in HFD mice were not associated with a change in either PKA signaling pathway (Figure 4F–G) or adipogenesis gene expression (Figure 4H–I). HFD induced a high circulating level of non-esterified fatty acids (FA) but not triglycerides (Figs. S4F-G). Thus, in the presence of high FA, the reduction of HSL and lipolysis likely contributed to the enlargement of adipocytes and increased body weight gain in mouse models (Figure 4J–K).

The administration of anti-MIF antibody *in vivo* neutralizes the effects of circulating and extracellular MIF [19]. In order to test the role of extracellular MIF on HSL, adipocyte mass and weight gain, we administered anti-MIF antibody for the last eight weeks of HFD. We found that immunoneutralization of MIF blunted the AMPK/JNK activation (Figure 4L) and inhibition of HSL (Figures 4M−N), while also partially reducing adipocyte hypertrophy (Figure 4O) and body weight gain (Figure 4P) during HFD feeding. These results further indicate that extracellular MIF mediates the activation of the AMPK/JNK signaling pathway in adipose tissue during HFD. Extracellular MIF has an important role in the downregulation of HSL and lipolysis and contributes to adipocyte hypertrophy and obesity during HFD.

As a consequence, anti-MIF also significantly decreased the high circulating FA levels associated with HFD (Fig. S5B), which reduced lipid accumulation in liver and skeletal muscle (Fig. S5C), promoted peripheral tissue insulin sensitivity (Figs. S5F-H) and limited whole-body insulin resistance (Fig. S5I). Interestingly, we found that the expression of the fatty acid oxidation regulator*, PPARα* in liver but not in skeletal muscle, was significantly reduced following HFD feeding; however, anti-MIF normalized liver *PPARα* during HFD (Figs. S5D-E). These findings suggest that MIF may inhibit fatty acid oxidation in liver which would indirectly increases hepatic lipid accumulation and contribute to the increase in circulating plasma FA levels. More interestingly, the neutralization of circulating MIF did not change HFD-upregulated expression of inflammatory factors, such as TNFα (*Tnfa*), IL1β (*II1b*) and IL6 (*II6*) in adipose tissue (Fig. S6), indicating that MIF induced metabolic changes were not associated with the induction of other inflammatory factors.

### 3.5 MIF receptor, CD74 is involved in the MIF/AMPK/JNK/HSL signaling pathway and obesity

**Figure 5:**
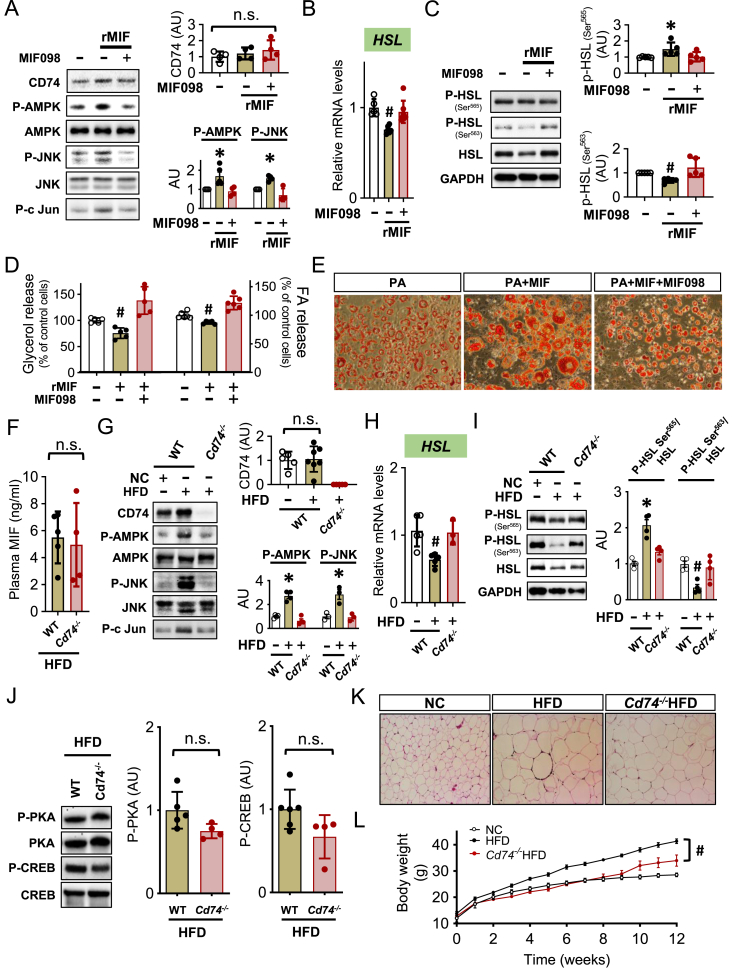
***The MIF receptor, CD74 is involved in the MIF/AMPK/JNK/HSL signaling pathway and obesity****.* 3T3-L1 adipocytes were incubated with vehicle, rMIF (400 mg/ml) or rMIF + MIF098 (10 μM) for 24 h. CD74, AMPK, JNK and c-Jun were evaluated with specific phospho- or total antibodies (A). *HSL* gene expression and activation were measured by qPCR or Western Blot (B and C). Release of glycerol and FA was quantified in (D). In the presence of high palmitic acid (PA), lipid droplet accumulation regulated by MIF and MIF098 was assessed with Oil red O staining (E). WT and *Cd74*^*−/−*^ mice were subjected to high fat diet (HFD) feeding for 12 weeks and their bloods were collected for the measurements of plasma MIF levels (F). Their adipose tissues were also harvested for the quantifications of CD74, AMPK, JNK, P-c Jun (G) and HSL expression and activation (H and I). In addition, phosphorylation of PKA and CREB was assessed in (J). HE staining in adipose tissues and body weight gain were monitored in WT and *Cd74*^*−/−*^ mice with or without HFD (K and L). A-D and G-I were analyzed by 1-way ANOVA, F and J were analyzed by 2-tailed Student's *t* test, and L was analyzed by multivariate (2-way) ANOVA. All data are presented as mean ± SD. ∗P ≤ 0.05 increase and ^#^P ≤ 0.05 reduction vs. other groups in A-D and G-I; vs. other groups in (G) to (I). ^#^P ≤ 0.05 reduction vs. HFD in (L). n.s. represents no significance.

The cognate MIF receptor CD74 is expressed in both 3T3-L1 adipocytes and adipose tissue (Figure 5A and G). In order to further assess the role of extracellular MIF on adipose signaling, we used the MIF inhibitor, MIF098 to block MIF binding with cell surface CD74 [30,31]. We found that MIF098 inhibited MIF-induced activation of the AMPK/JNK signaling pathway (Figure 5A) in 3T3-L1 adipocytes and blocked the effects of MIF to reduce HSL expression and activation (Figure 5B–C). In addition, MIF098 prevented the reduction of adipose glycerol content and fatty acid release induced by MIF (Figure 5D). In the presence of PA, MIF098 inhibited MIF-induced lipid accumulation in adipocytes (Figure 5E).

In order to examine the role of CD74 *in vivo*, we performed parallel experiments in CD74 deficient mice (*Cd74*^*−/−*^) subjected to HFD. These experiments showed that lack of CD74 blocked the ability of HFD to trigger AMPK/JNK activation (Figure 5G) or to inhibit HSL (Figure 5H–I). This could not be explained by differences in plasma MIF levels, which were similar in WT and *Cd74*^*−/−*^ mice during HFD (Figure 5F). The phosphorylation of both PKA and its downstream target, CREB was unchanged (Figure 5J). As with neutralizing MIF, CD74 knockout also significantly reduced adipocyte hypertrophy (Figure 5K) and body weight gain (Figure 5L) following HFD feeding, suggesting that CD74 may be a key regulator of the MIF/AMPK/JNK/HSL pathway during obesity.

### 3.6 Deficiency of intracellular MIF inhibits HSL expression through JNK but independent of AMPK

**Figure 6:**
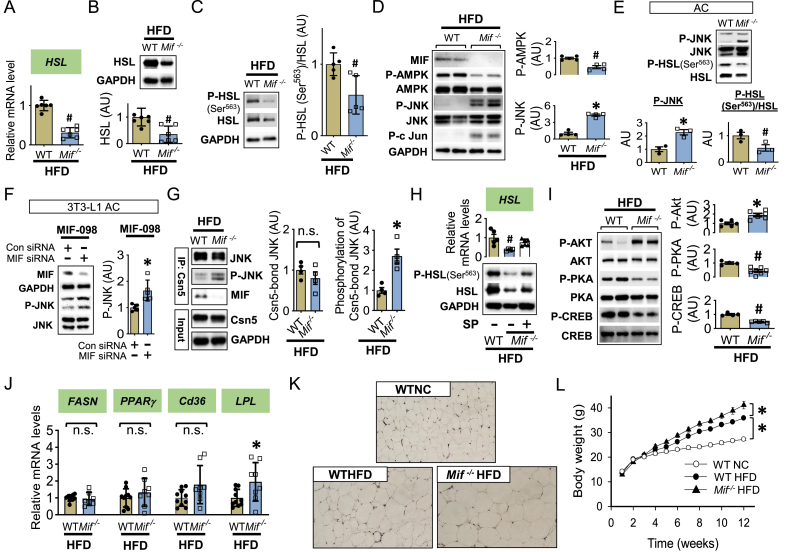
***Deficiency of intracellular MIF inhibits HSL expression through JNK but independent of AMPK***. WT and *Mif*^*−/−*^ mice at 3 weeks were fed with normal chow (NC) or high fat diet (HFD) for 12 weeks. The gene (A) and protein (B) expression of HSL in adipose tissue was quantified with qPCR and western blot, respectively. The phosphorylation of HSL at Ser^563^ (C), AMPK and JNK phosphorylation (D) and MIF levels (D) were also examined. Adipocytes (AC) were isolated from WT and *Mif*^*−/−*^ adipose tissue and the phosphorylation of JNK and HSL Ser^563^/HSL ratio were evaluated by western blot (E). In 3T3-L1 adipocytes (3T3-L1 AC), following knockdown of MIF with MIF siRNA, MIF and JNK phosphorylation in the presence of MIF inhibitor, MIF098 (10 μM) were evaluated by western blot in (F). The interaction among JNK, MIF and Csn5 was determined by co-immunoprecipitation (G). Phosphorylation of Csn5-bond JNK was quantified by western blot (G). Adipose tissues were isolated from WT and *Mif*^*−/−*^ mice following HFD and they were cultured in physiological KREB solution at 37 °C for 24 h in the absence or presence of SP600125. *HSL* gene and protein expression were detected by qPCR and western blot (H). The phosphorylation of Akt and PKA was examined in (I). Adipogenesis gene expressions, such as *FASN*, *PPARγ*, *C36* and *LPL* were quantified by qPCR (J). Hematoxylin–eosin (HE) staining was performed to identify adipocyte hypertrophy in (K) and body weight gain was monitored in (L) weekly. H was analyzed by 1-way ANOVA, L was analyzed by multivariate (2-way) ANOVA, and the rest of bar graphs were analyzed by 2-tailed Student's *t* test. All data are presented as mean ± SD. ∗P ≤ 0.05 increase and ^#^P ≤ 0.05 reduction vs. WT or WTHFD. n.s. represents no significance.

In order to assess the overall role of both extracellular and intracellular MIF in regulating HSL and adipose lipid metabolism, we next studied *Mif*^*−/−*^ mice with global genetic deletion of MIF. Surprisedly, *Mif*^*−/−*^ mice administered a high fat diet demonstrated a reduction in HSL expression and activation in adipose tissue compared to WT (Figure 6A–C), suggesting that intracellular MIF may have an important role in regulating HSL expression. As expected, *Mif*^*−/−*^ mice demonstrated a reduced phosphorylation of AMPK (Figure 6D) compared to WT mice, with loss of action of extracellular MIF to activate AMPK. However, this reduction of AMPK phosphorylation was associated with a loss of inhibitory phosphorylation of HSL at Ser^565^ (Figure S7). Surprisingly, *Mif*^*−/−*^ mice demonstrated an increase in JNK phosphorylation in adipose tissue (Figure 6D). These results suggest that intracellular MIF might have an opposite effect to suppress JNK signaling, which would oppose the effect of extracellular MIF to activate JNK signaling.

We also found that adipocytes isolated from *Mif*^*−/−*^ mice demonstrated enhanced JNK phosphorylation and reduced HSL activation compared to WT (Figure 6E). To test how the reduction of intracellular MIF regulates JNK activation, we partially deleted MIF expression in cultured 3T3-L1 adipocytes by MIF siRNA. We also treated these cells with MIF-098 to block the extracellular effect of MIF. We found that intracellular MIF reduction following MIF siRNA treatment led to overactivation of JNK phosphorylation (Figure 6F). These findings further support the conclusion that intracellular MIF deficiency contributes to JNK activation in adipocytes and thus has an opposing action to extracellular MIF.

Previous studies indicate that intracellular MIF inhibits JNK activation by directly binding to the transcription factor Csn5 [14]. We immunoprecipitated Csn5 and found that it bound both MIF and JNK in adipose tissue from HFD WT mice. We hypothesized that the formation of a MIF, Csn5 and JNK complex might downregulate JNK activation in WT mice, and this inhibitory effect of intracellular MIF might be lost in *Mif*^*−/−*^ mice. We found that in the absence of MIF, JNK phosphorylation was indeed augmented (Figure 6G), although the binding between Csn5 and JNK was intact in *Mif*^*−/−*^ mice (Figure 6G). To further test whether the overactivation of JNK in *Mif*^*−/−*^ mice contributed to the downregulation of HSL expression, we incubated adipose tissues from WT and *Mif*^*−/−*^ mice with the JNK inhibitor, SP600125 (Figure 6H). We found that SP treatment reversed both overactivated JNK and the reduction in HSL expression and activation (Figure 6H).

We then assessed the effects of the loss of MIF in modulating plasma lipids during HFD. MIF neutralizing antibody reduced circulating FA concentrations in WT mice following HFD (Fig. S6B), however, this effect on FA was not observed in the *Mif*^−/−^ mouse model (Fig. S8A). In addition, *Mif*^*−/−*^ mice showed significantly increased plasma triglyceride levels (Fig. S8B), indicated more severe hyperlipidemia in *Mif*^*−/−*^ compared to WT mice. WT mice on a HFD had reduced Akt phosphorylation in liver, adipose tissue and skeletal muscle (Figs. S4C-E), which was exacerbated in the livers of *Mif*^*−/−*^ mice on a HFD (Fig. S8C), suggesting worsening hepatic insulin resistance associated with MIF deletion. In adipose tissue, *Mif*^*−/−*^ mice had a higher level of Akt phosphorylation (Figure 6I) when compared to WT following HFD. The increased Akt phosphorylation was associated with attenuated PKA signaling pathway (Figure 6I), suggesting that MIF deficiency might downregulate HSL activation through an Akt/PKA signaling pathway. In contrast, we observed equivalent phosphorylation levels of Akt in skeletal muscle in *Mif*^*−/−*^ compared with WT mice on a HFD (Fig. S8D).

Thus, in *Mif*^*−/−*^ mice, the inhibition of adipose HSL is due to the overlapping effects of: (1) decreased *HSL* gene expression and (2) attenuated HSL activation by the AKT/PKA signaling pathway. Accordingly, the levels of HSL in MIF deficient mice are lower than those in WT mice (Figure 6C). By screening adipogenesis genes, we also found that MIF deletion increased *LPL* gene expression in adipose tissue (Figure 6J) which may also contribute to the development of adipocyte hypertrophy and obesity. Indeed, *Mif*^*−/−*^ mice had more severe adipocyte hypertrophy compared to WT mice following high fat diet feeding (Figure 6K), which was associated with more weight gain (Figure 6L) and whole-body insulin resistance (Fig. S9).

## 4 Discussion

Lipolysis is accelerated by inflammatory cytokines, such as TNF-α and IL-4 [5,6], and this process may be related to the activation of HSL [6]. However, the current results show that the cytokine, MIF, expressed upstream of these cytokines [32,33], inhibits HSL and lipolysis in adipose tissue, thereby exacerbating adipocyte hypertrophy and contributing to the development of obesity. MIF is released from pre-formed storage pools in response to stimuli and has autocrine-paracrine actions to regulate cellular function and metabolism by binding to the cell membrane receptor, CD74. We show that extracellular MIF has autocrine actions to downregulate HSL through activation of AMPK/JNK signaling after binding to CD74. Mice overexpressing MIF, as well as WT mice fed a HFD that caused high circulating MIF levels, showed suppression of HSL, which was associated with the development of obesity. Blocking the extracellular action of MIF by a neutralizing anti-MIF antibody significantly prevented the development of HFD-induced obesity which is associated with reduced adipose AMPK/JNK signaling and reversed HSL in HFD mice, further suggesting a role for extracellular MIF in downregulating HSL. Unexpectedly, however, mice with MIF global deletion also had reduced HSL activation and expression in adipose tissue. This finding led us to investigate the role of a potential distinct MIF regulatory pathway, in which intracellular MIF downregulates JNK activation by binding to the intracellular protein, Jab1/Csn5 [14]. Knockdown of MIF had additional effects after blockade of the extracellular pathway, which recapitulated changes in global *Mif*^*−/−*^ mice. Global deletion of MIF led to hyperactivation of JNK, resulting in reduced HSL gene and protein expression. MIF deficiency also increased Akt and downregulated PKA signaling compared to WT mice fed a HFD. Inhibition of PKA downregulated HSL activation and enhanced adipocyte hypertrophy. Thus, our present data suggest that both intracellular and extracellular MIF have opposing effects to regulate HSL, but the extracellular actions predominate to downregulate HSL and exacerbate the development of obesity during HFD.

Metabolic dysfunction, including obesity and insulin resistance, is associated with reduced HSL expression in adipose tissue [3]. *HSL* mRNA expression is diminished in visceral adipose tissues from obese subjects and is strongly correlated with human adipocyte size and plasma insulin concentrations [34]. Interestingly, insulin resistance is inversely associated with adipose HSL expression but not with adipocyte size and body composition [3]. Together, these data suggest that regulation of HSL in adipose tissue may contribute to adipocyte hypertrophy and the development of insulin resistance. However, the underlying mechanisms by which HSL is regulated are largely unknown. Our present study is the first to investigate the regulation of adipose HSL expression by the immune cytokine MIF. In metabolic disorders, MIF may be released from circulating monocytes and adipose tissue, leading to high plasma MIF levels [7]. We found that extracellular MIF inhibits the expression and activation of HSL and lipolysis in adipocytes by binding to its cell membrane receptor, CD74. This effect of MIF leads to augmented adipocyte hypertrophy in the presence of high fatty acid or a high fat diet. Interestingly, this effect was independent of alterations in the expression of adipogenesis genes. Furthermore, blocking the extracellular action of MIF by a neutralizing MIF antibody significantly reduced obesity and insulin resistance in HFD mice, suggesting a critical role for extracellular MIF in regulating metabolism through downregulation of HSL.

We found that extracellular MIF downregulates HSL activation and subsequent lipolysis in adipocytes by activating AMPK. MIF is known to stimulate AMPK activation in different cell types, such as cardiomyocytes [11], neurons [10] and liver cells [35] through CD74. Previous studies indicated that AMPK phosphorylates HSL at Ser^565^ and inhibits HSL activity [28]. In this study, we found that AMPK activation induced by both AICAR and MIF also downregulates HSL gene and protein expression. The transcriptional effect of AMPK on HSL seems to be critical with respect to the regulation of lipolysis. Interestingly, AMPK activation did not affect expression of the lipolytic enzyme ATGL, suggesting a selective effect on HSL to inhibit lipolysis. MIF is also known to activate JNK in cardiomyocytes and immune cells during myocardial ischemia-reperfusion [36] and inflammation [37], respectively. Here, we show that MIF treatment stimulates JNK phosphorylation through AMPK in adipocytes. Furthermore, AMPK/JNK signaling appears to be regulated by extracellular MIF (through CD74) and has an important transcriptional regulatory effect on HSL that inhibits lipolysis.

Although blocking the extracellular action of MIF by a neutralizing MIF antibody successfully reversed HSL down-regulation and reduced obesity in HFD mice, MIF transgenic knockout mice with global loss of MIF action had an opposing effect. Previous studies indicated that intracellular MIF has an inhibitory effect on the intracellular protein, Csn5 which normally activates JNK activity and enhances phospho-c Jun levels [14]. Although our data indicate that intracellular MIF did not affect the binding between Csn5 and JNK, it inhibited Csn5-mediated JNK phosphorylation and activation. Thus, after global deletion of MIF in *Mif*^*−/−*^ mice, the loss of intracellular inhibition of adipose Csn5-mediated JNK phosphorylation, augmented JNK phosphorylation compared to WT following high fat diet feeding. Thus, despite loss of CD74-AMPK activation resulting from the absence of extracellular MIF, intracellular JNK activation remained elevated and *HSL* gene expression was still inhibited in global *Mif*^*−/−*^ mice. It should be noted that this mechanism was only observed during global MIF deletion and is unlikely to occur under more physiological conditions. This might suggest that the enhanced body weight gain observed in global *Mif*^*−/−*^ mice during HFD [38], was probably due to the activation of JNK/HSL signaling pathway in mice with global deletion of intracellular MIF.

Normally, MIF attenuates insulin sensitivity by inducing insulin receptor substrate (IRS) serine phosphorylation in adipocytes [25]. Although whole-body insulin resistance in *Mif*^*−/−*^ mice fed a high fat diet is unchanged or even worse due to exacerbated insulin resistance in liver, they have improved adipose Akt phosphorylation compared to WT. Akt-downregulated PKA may contribute to the inhibition of HSL in the *Mif*^*−/−*^ mice. In addition, the development of obesity is generally regulated by increased adipogenesis and/or decreased lipolysis. Extracellular MIF inhibits HSL and lipolysis without associated changes in adipogenesis gene expression. However, with a reduction in HSL, the deficiency of intracellular MIF leads to an upregulation of lipoprotein lipase (LPL) that may augment adipogenesis. Thus, *Mif*^*−/−*^ mice have more severe obesity compared to WT following high fat diet feeding. Whether this effect results primarily from intracellular or extracellular MIF is currently unknown.

It should be noted that mice [39] with HSL knockout and human subjects with HSL mutations [40] have reduced lipolysis in adipose tissue, but do not develop obesity [41]. Interestingly, mice with global HSL knockout have enlarged adipocytes in white and brown adipose tissues despite the lack of overall obesity [42]. In contrast, our present findings suggest that extracellular MIF contributes to both HSL downregulation and adipocyte hypertrophy and obesity. It is possible that HSL mutations during development cause some degree of loss of adipose cell number and lipodystrophy, which does not occur with post-developmental MIF-mediated loss of HSL during HFD. We also did not observe any change in markers of lipogenetic genes with HFD. In addition, MIF neutralization by anti-MIF antibody significantly reversed HSL, but not the expression of *TNFα*, *IL-1β* and *IL6*, suggesting that MIF regulated HSL and body weight independent of changes in adipose tissue inflammation.

MIF has different regulatory effects on different organs that express CD74. In the heart, during ischemia-reperfusion, cardiomyocyte-derived MIF promotes glucose metabolism by activating AMPK to compensate for energy deprivation and prevent cardiac injury [11]. In the liver, MIF stimulated AMPK counteracts the development of liver fibrosis [35]. However, MIF derived from adipose tissue has been suggested to be detrimental to the development of metabolic dysfunction [9,43]. High fat diet induced adipose MIF release is positively associated with insulin resistance in the presence or absence of adipose inflammation [44,45]. MIF attenuates insulin signaling only in adipocytes but not in liver or skeletal muscle cells [10]. Our present study also indicated that MIF inhibits HSL by activating the AMPK/JNK signaling pathway. The diverse effects of MIF are most likely cell specific, governing in large part by CD74 expression and its coupling to intracellular signaling intermediates. There is also growing evidence that MIF may have both intracellular and extracellular effects on the same cellular signaling targets. For example, the intracellular effect of MIF in inhibiting the JNK signaling pathway was observed in cancer cells [14], but the extracellular effect of MIF in activating JNK through the CD74 receptor was reported in T cells and fibroblasts [37]. Interestingly, our present data show for the first time that both intracellular and extracellular MIF are present in adipocytes and appear to have opposing effects on HSL and lipolysis. JNK is a key mediator of both effects. Previous studies have showed that glucocorticoids can bind to membrane glucocorticoid receptors or cytosolic glucocorticoid receptors thereby exerting multiple effects in regulating inflammation [46]. Serotonin has extracellular effects in regulating heart valve development, but it also has intracellular effects on heart valve remodeling during disease [47]. Therefore, MIF may be one of the secreted factors that exhibit both extracellular and intracellular effects under physiological and pathological conditions in the human body.

Although intracellular regulation of JNK by MIF is only revealed under the extreme condition of complete MIF absence, strategies to selectively inhibit extracellular MIF with neutralizing antibody or small molecule antagonism of MIF interaction with cell surface CD74 appear to have more beneficial metabolic actions. The strategy to develop CD74 inhibitors for the treatment of metabolic dysfunction may be most effective in subjects with high MIF expression, for instance due to their genetic predisposition for a high expression MIF allele [10].

## Author contributions

L. C. and L. L performed the major experiments. Y. H., Y. Q. and H. Z. participated in animal studies. D. C., H. T., H. W., S. W., S. L., L. L. and B. R. contributed intellectually to data analysis and manuscript editing. T. L. was involved in the preparation of recombinant MIF proteins. L. Y. and R. B. provided overall scientific support for the research project and D. Q. designed and managed the research. All authors read and approved the final manuscript. D. Q. is the guarantor of this work and, as such, had full access to all the data in the study and takes responsibility for the integrity of the data and the accuracy of the data analysis.

## Funding

This study was supported by National Sciences and Engineering Research Council of Canada (NSERC: RGPIN-2017-04542) and 10.13039/501100000024Canadian Institutes of Health Research (10.13039/501100000024CIHR Project Grant: PJT-156116) for Dr. Qi, NIH R01-AR-078334 for Dr. Bucala, and China Scholarship Council Fellowships for Liujun Chen, Yiheng Huang and Lisha Li.

## Declaration of competing interest

All the authors declare that no potential conflicts of interest relevant to this article.

## Data Availability

Data will be made available on request.
