# Supplementary material for: Extracellular macrophage migration inhibitory factor (MIF) downregulates adipose hormone-sensitive lipase (HSL) and contributes to obesity
Source: Mol Metab. 2023 Nov 5;79:101834. doi: 10.1016/j.molmet.2023.101834 (PMC10700858; doi:10.1016/j.molmet.2023.101834)
Supplement: Multimedia component 1 [file mmc1.pdf]

Table. S1. List of PCR primer sequences

Gene Name	Sequences (5'–3')
ATGL	GAG ACC AAG TGG AAC ATC<br>GTA GAT GTG AGT GGC GTT
HSL	CAG AAG GCA CTA GGC GTG ATG<br>GGG CTT GCG TCC ACT TAG TTC
PPARg	ACA GAC AAA TCA CCA TTC GT<br>CTC TTT GCT CTG CTC CTG
FASN	CAT GAC CTC GTG ATG AAC GTGT<br>CGG GTG AGG ACG TTT ACA AAG
LPL	AGA GCC AAA AGA AGC AG<br>GGC AGA GTG AAT GGG AT
CD36	TTG AAA AGT CTC GGA CAT TGA T<br>TCA GAT CCG AAC ACA GCG TA
PPARα	TGG TGT TCG CAG CTG TTT TG<br>AGA TAC GCC CAA ATG CAC CA
CPT-1	ACT CCG CTC GCT CAT TCC G<br>TGT TTG AGG GCT TCA TGG CT
Tnfa	CAG GCG GTG CCT ATG TCT C<br>CGA TCA CCC CGA AGT TCA GTA G
Il1b	TGG TGT GTG ACG TTC CCA TT<br>CAG CAC GAG GCT TTT TTG TTG
Il6	GAG GAT ACC ACT CCC AAC AGA CC<br>AAG TGC ATC ATC GTT GTT CAT ACA
GAPDH	ATG TGT CCG TCG TGG ATC TGA<br>TGC CTG CTT CAC CAC CTT CTT

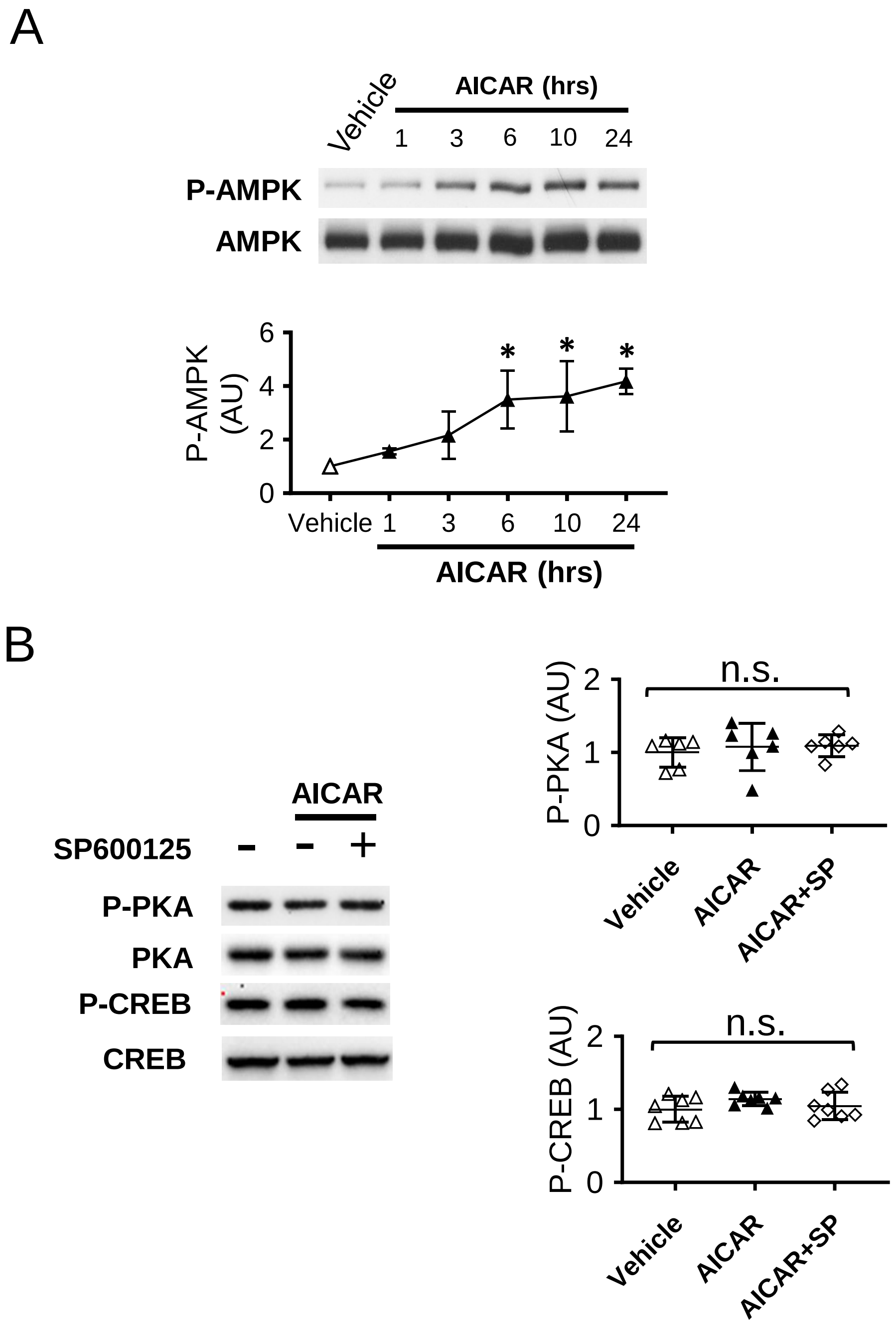

Fig. S1. The effects of AICAR on AMPK and PKA/CREB. AICAR stimulates AMPK phosphorylation in a time-dependent manner (A) without affecting PKA and CREB phosphorylation (B). JNK inhibition by SP600125 does not change the PKA/CREB signaling pathway (B). All data are presented as mean ± SD. A and B were analyzed by 1-way ANOVA. *P ≤ 0.05 increase vs. Vehicle. n.s. represents no significance.

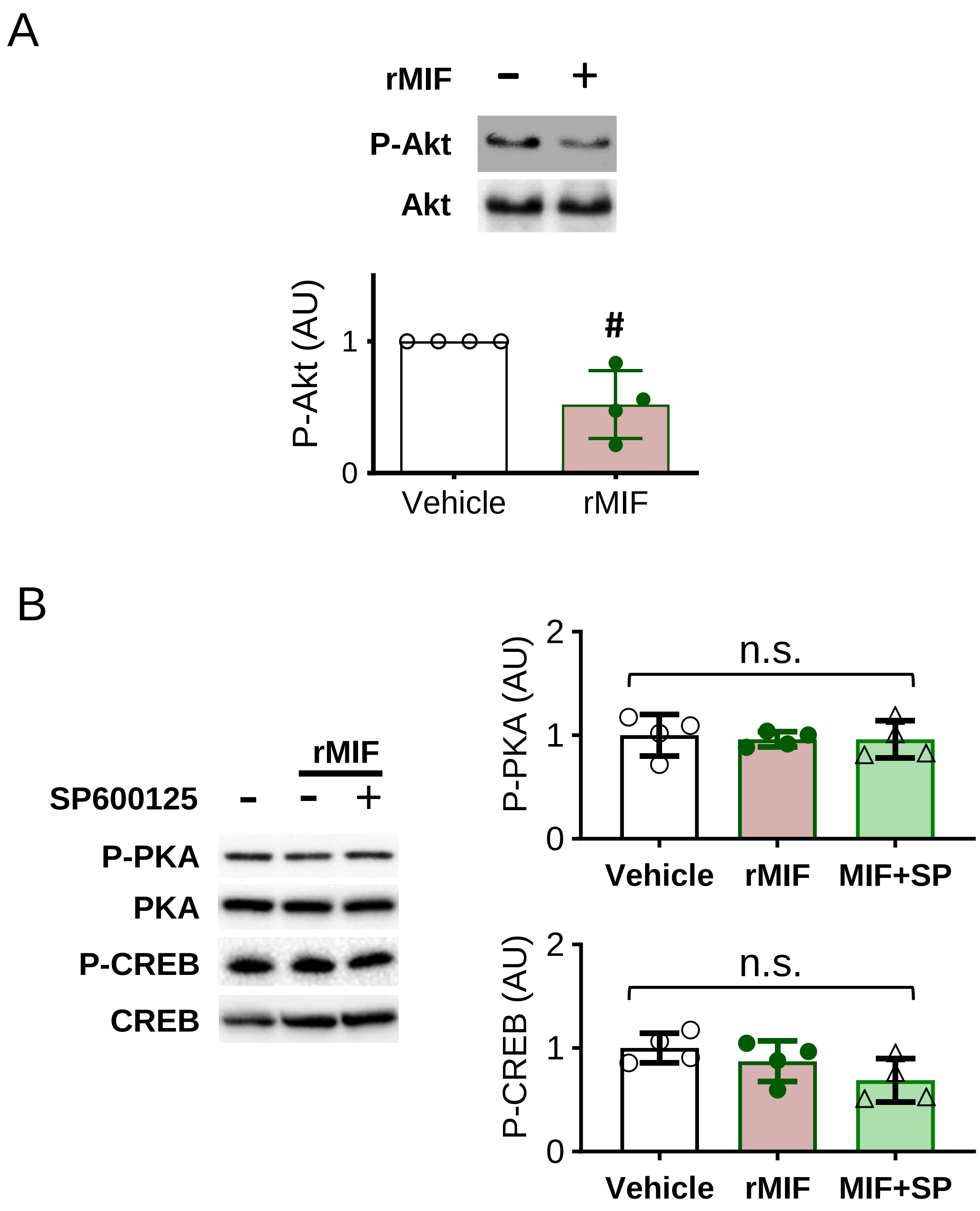

Fig. S2. The effects of MIF on phosphorylation of Akt and PKA. Vehicle or 400ng/ml of recombinant mouse MIF was incubated with 3T3-L1 differentiated adipocytes for 24 hours. Akt (A) and PKA (B) phosphorylation was evaluated by western blot. SP600125 was also incubated with MIF to investigate whether the inhibition of JNK affects PKA and CREB phosphorylation (B). All data are presented as mean ± SD. A was analyzed by 2-tailed Student's *t* test and B was analyzed by 1-way ANOVA. $^{\#}P \leq 0.05$ reduction vs. Vehicle. n.s. represents no significance.

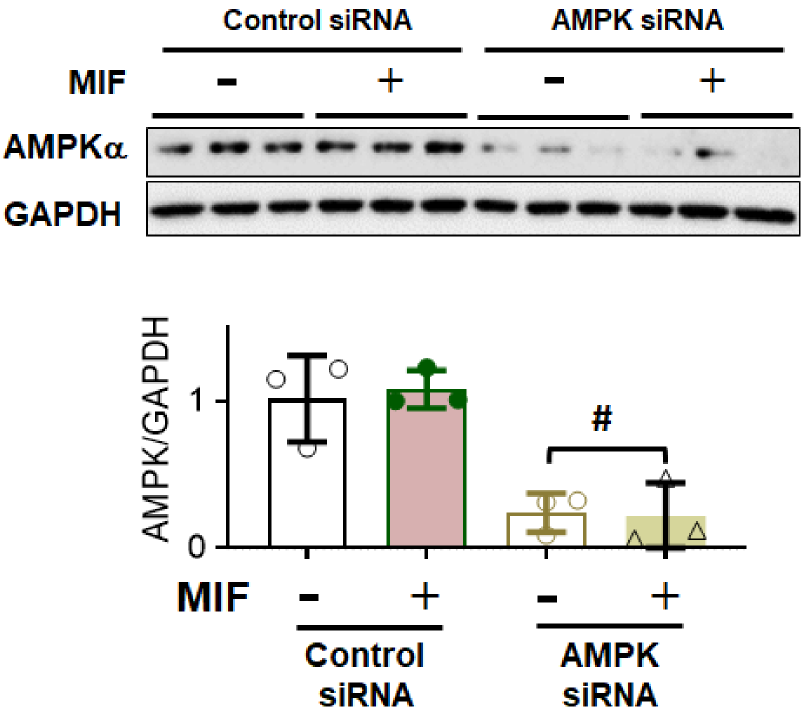

Fig. S3. ***AMPK α1* and *α2* subunits were knocked by *siRNA*.** All data are presented as mean ± SD. The data were analyzed by 1-way ANOVA. #P ≤ 0.05 reduction vs. Control siRNA.

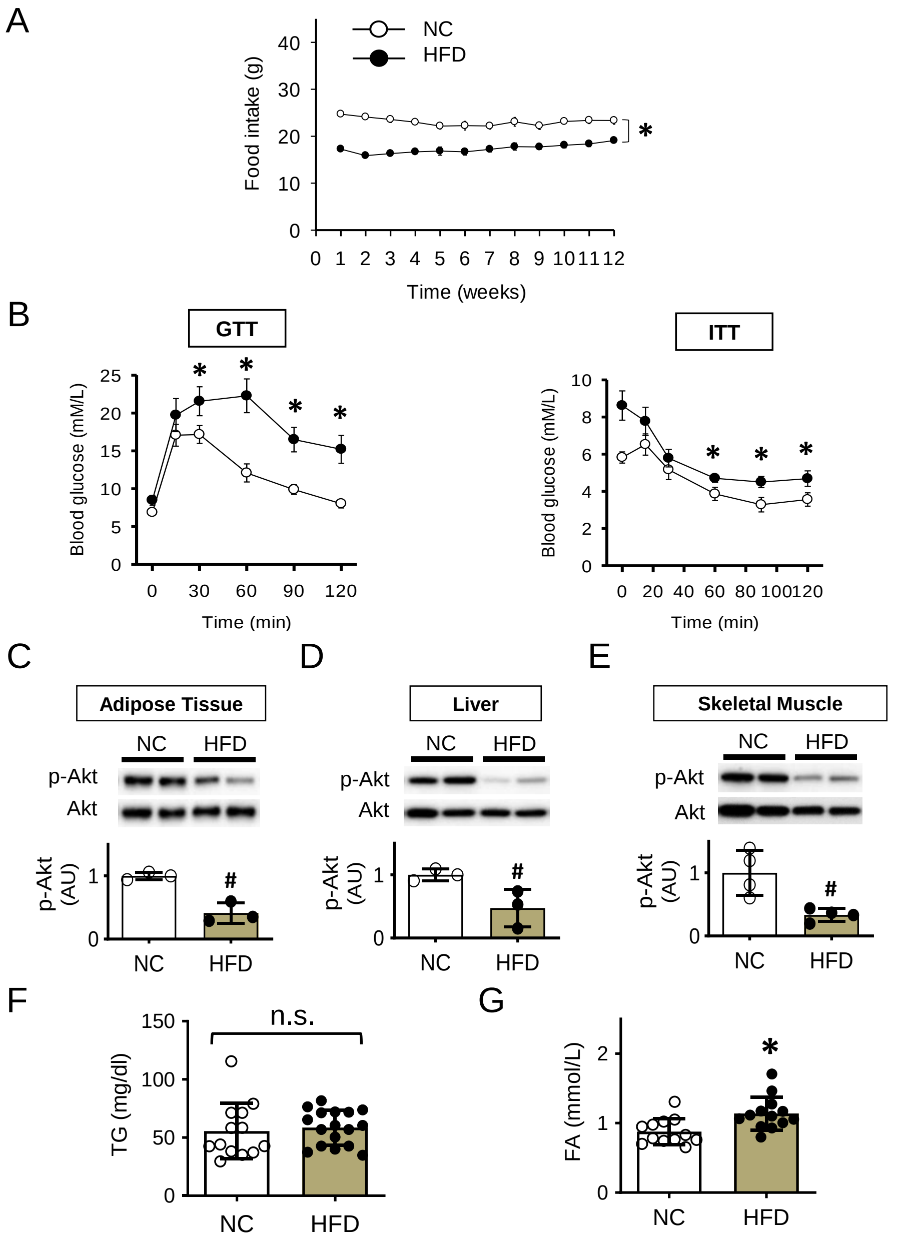

Fig. S4. High caloric diet induces insulin resistance and hyperlipidemia. C57BL/6 mice (3 weeks) were fed with normal chow (NC) or high caloric diet (HFD) for 12 weeks. The food intake was monitored in (A). Whole-body insulin resistance was evaluated by glucose tolerance test (GTT) and insulin tolerance test (ITT) (B). Insulin stimulated Akt phosphorylation in peripheral tissues was measured by western blot (C to E). Serum triglyceride (TG) (F) and fatty acid (FA) (G) levels were examined by commercial kits. N= 3-11 each animal group. All data are presented as mean ± SD. A and B were analyzed by multivariate (2-way) ANOVA and C-G were analyzed by 2-tailed Student's *t* test. *P ≤ 0.05 increase and #P ≤ 0.05 reduction vs. NC. n.s. represents no significance.

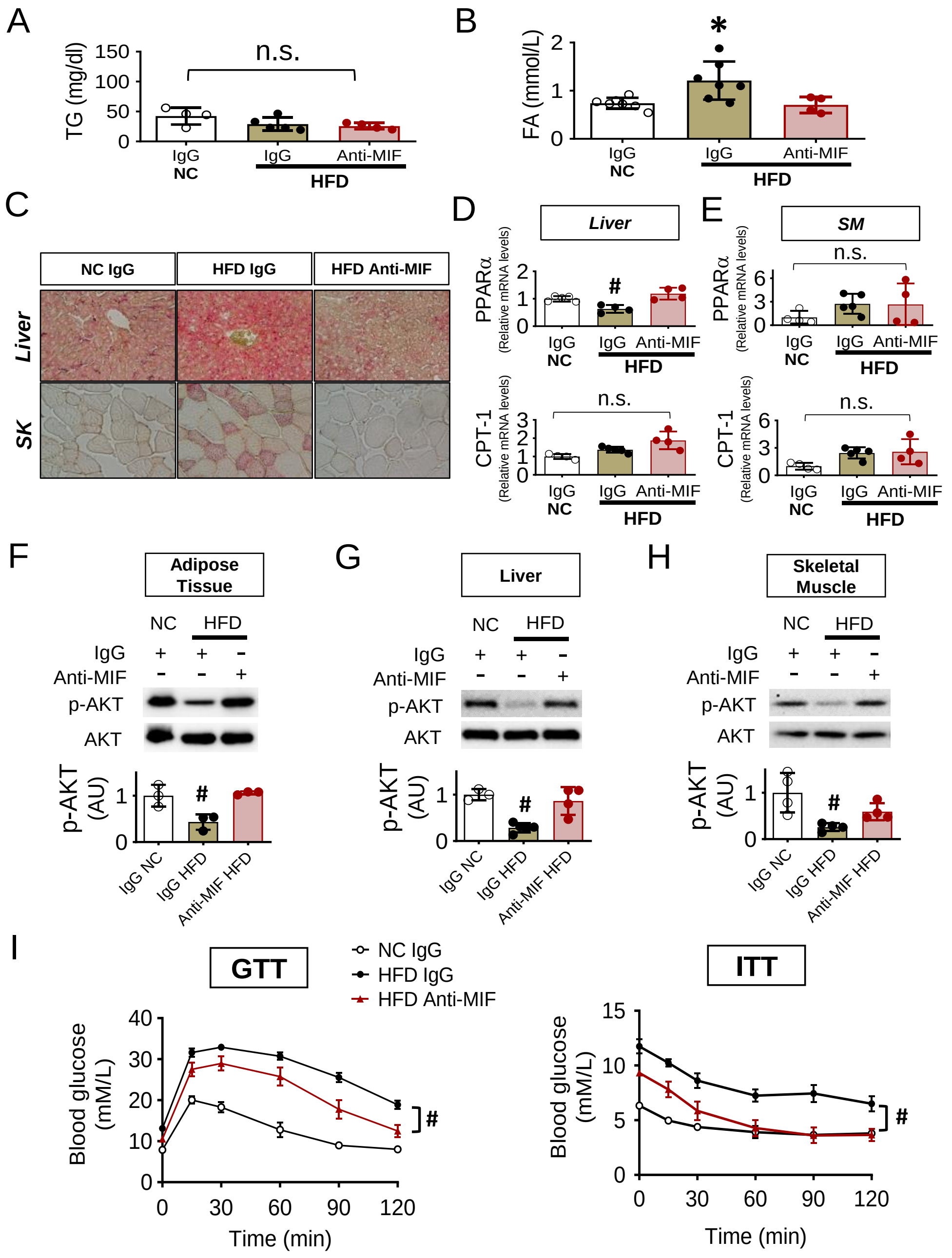

Fig. S5. Neutralization of MIF reverses HFD induced metabolic dysfunction. C57BL/6 mice (3 weeks) were fed with normal chow (NC) or high caloric diet (HFD) with IgG or anti-MIF antibody injection (twice per week) for 12 weeks. Serum triglyceride (TG) (A) and fatty acid (FA) (B) levels were examined, and Oil red O staining was performed to detect lipid accumulation in the liver and skeletal muscle (C). The gene expression of *PPARα* and *CPT-1* was quantified in liver and skeletal muscle by qPCR (D and E). Insulin stimulated Akt phosphorylation in peripheral tissues was measured by western blot (F to H). Whole-body insulin resistance was evaluated by glucose tolerance test (GTT) and insulin tolerance test (ITT) (I). N= 3-7 for each animal group. All data are presented as mean ± SD. A-H were analyzed by 1-way ANOVA and I was analyzed by multivariate (2-way) ANOVA. *P ≤ 0.05 increase vs. other groups in (B); $^{\#}$P ≤ 0.05 reduction vs. other groups in (D), (F) to (H); and $^{\#}$P ≤ 0.05 reduction vs. HFD IgG in (I). n.s. represents no significance.

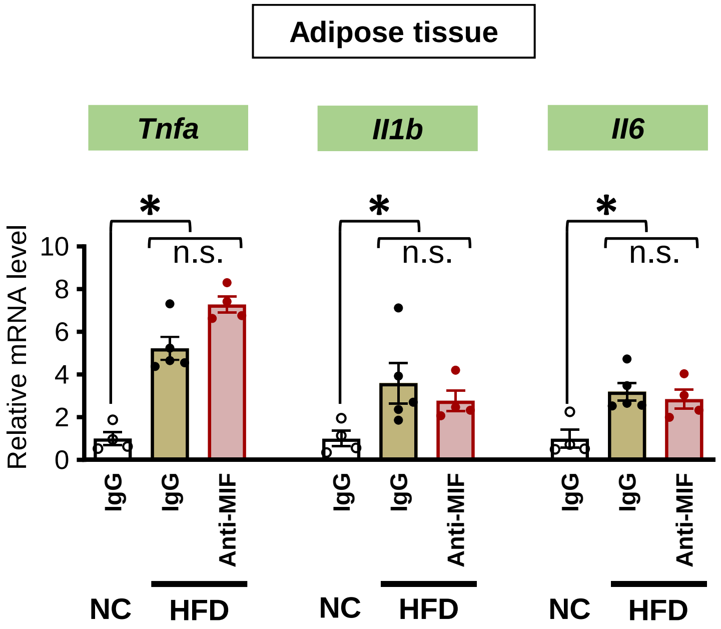

Fig. S6. Neutralization of MIF did not affect HFD induced inflammatory gene expression. C57BL/6 mice (3 weeks) were fed with normal chow (NC) or high caloric diet (HFD) with IgG or anti-MIF antibody injection (twice per week) for 12 weeks. The gene expression of inflammatory factors, *Tnfa*, *Il1b* and *Il6* was quantified by qPCR. N= 4-5 each animal group. The data are presented as mean ± SD and analyzed by 1-way ANOVA. *P<0.05 increase vs. IgG. n.s. represents no significance.

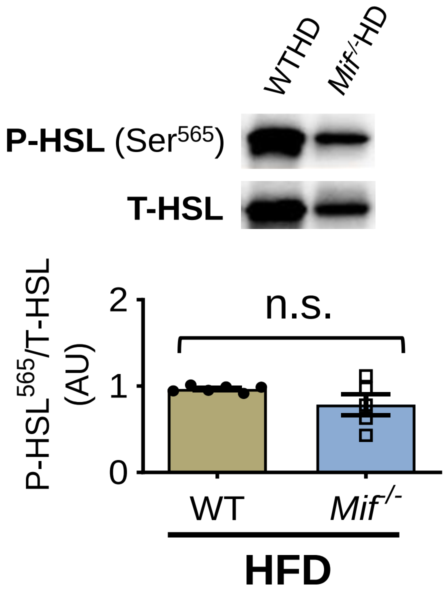

Fig. S7. *Mif*$^{-/-}$ mice have unchanged HSL phosphorylation at the site of Ser^{565} following HFD compared to WT. WT and *Mif*$^{-/-}$ mice (3 weeks) were fed with high caloric diet (HFD) for 12 weeks. HSL phosphorylation at the site of Ser^{565} was quantified by western blot. N= 5-6 for each animal group. The data are presented as mean ± SD and analyzed by 2-tailed Student's *t* test. n.s. represents no significance.

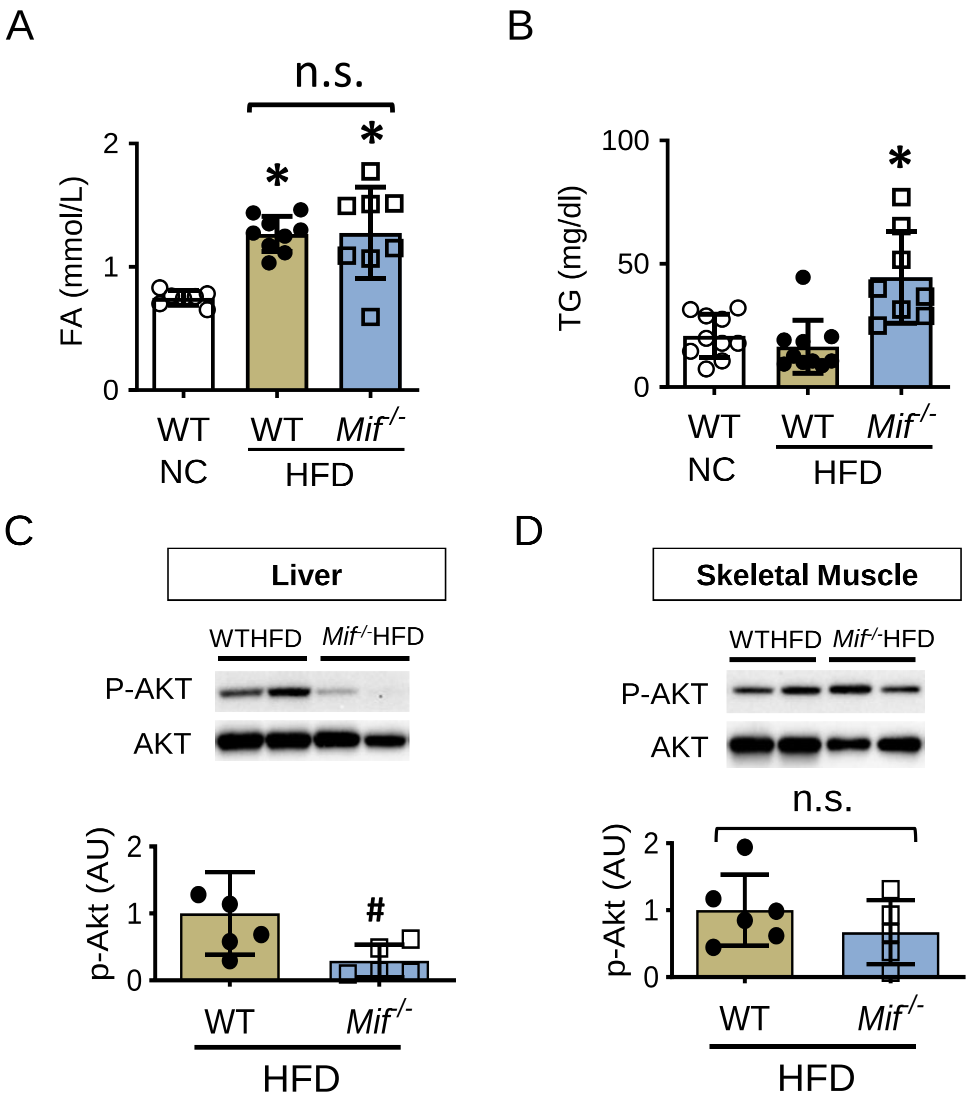

Fig. S8. *Mif*$^{-/-}$ mice have exacerbated hyperlipidemia and tissue specific insulin resistance in liver and skeletal muscle. WT and *Mif*$^{-/-}$ mice (3 weeks) were fed with high caloric diet (HFD) for 12 weeks. Serum triglyceride (TG) (A) and fatty acid (FA) (B), and Akt phosphorylation in liver (C) and skeletal muscle (D) were quantified. N= 5-10 for each animal group. All data are presented as mean ± SD. A and B were analyzed by 1-way ANOVA, and C and D was analyzed by 2-tailed Student's *t* test. *P ≤ 0.05 increase vs. WT NC in (A) and (B); and $^{\#}$P ≤ 0.05 reduction vs. WT HFD. n.s. represents no significance.

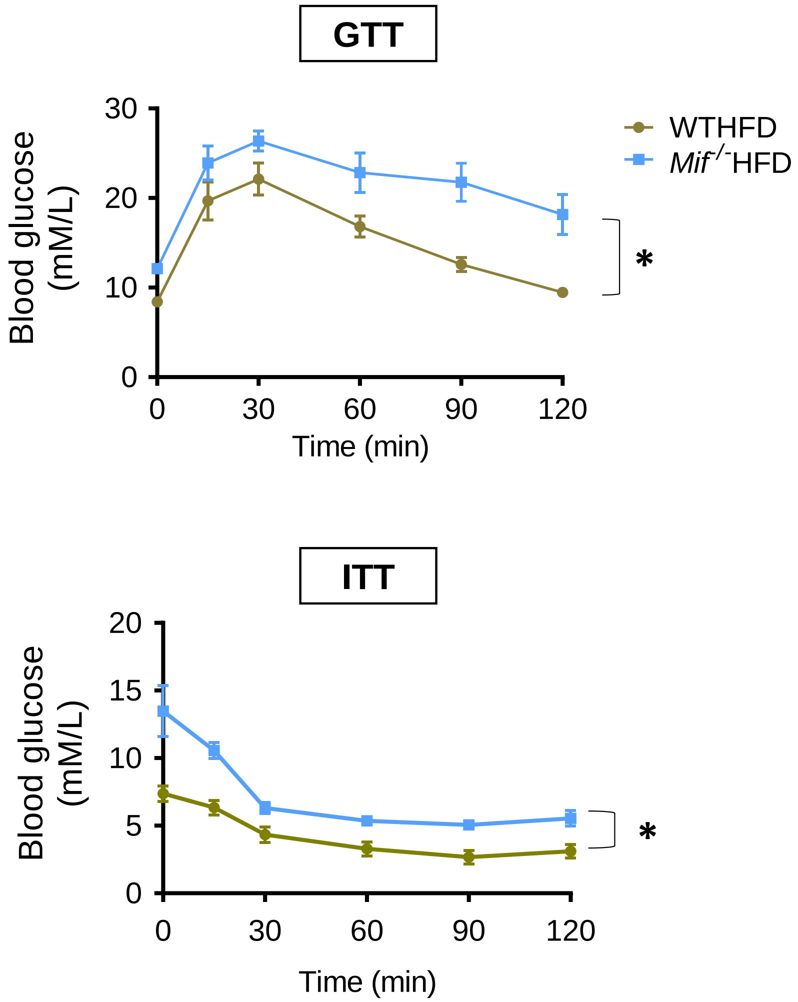

Fig. S9. *Mif$^{-/-}$* mice have more severe whole-body insulin resistance compared to WT following HFD. WT and *Mif$^{-/-}$* mice (3 weeks) were fed with high caloric diet (HFD) for 12 weeks. GTT and ITT (D) were then quantified by i.p. injection of high glucose or insulin. N= 6 for each animal group. All data are presented as mean ± SD. The data were analyzed by multivariate (2-way) ANOVA. *P ≤ 0.05 increase vs. WT HFD.
